# HERV-K(HML-2), the Best Preserved Family of HERVs: Endogenization, Expression, and Implications in Health and Disease

**DOI:** 10.3389/fonc.2013.00246

**Published:** 2013-09-20

**Authors:** Oliver Hohn, Kirsten Hanke, Norbert Bannert

**Affiliations:** ^1^Division for HIV and Other Retroviruses, Robert Koch Institute, Berlin, Germany

**Keywords:** human endogenous retrovirus, HERV-K, replication, Rec, cancer

## Abstract

Retroviruses that have the ability to infect germ line cells can become an integral and inherited part of the host genome. About 8% of the human chromosomal DNA consists of sequences derived from infections by retroviruses that presumably circulated 2–40 millions of years ago, and some elements are actually much older. Post-insertional recombinations, deletions, and mutations have rendered all known human endogenous retroviruses (HERVs) non-infectious. However some, particularly the most recently acquired proviruses of the HERV-K(HML-2) family, can expresses viral proteins and produce viral particles. In this review we will first discuss the major aspects of the endogenization process and peculiarities of the different HERV-K families. We will then focus on the genes and proteins encoded by HERV-K(HML-2) as well as inactivation of these proviruses by postinsertional mutations and their inhibition by antiretroviral factors. After describing the evolutionary interplay between host and endogenous retrovirus we will delve deeper into the currently limited understanding of HERV-K and its possible association with disease, particularly tumorigenesis.

## Introduction

Human chromosomes contain numerous retroviral sequences that form part of our genetic legacy. In total, human endogenous retroviruses (HERVs) sequences account for ∼8% of our genome. With more than 500,000 elements, there are over 20-fold more HERV sequences than there are human genes. These elements typically have their origin in ancient infections of germ cells by exogenous retroviruses that circulated ∼2–40 million years ago. The human genome and the genomes of other vertebrate species that contain endogenous retroviruses can therefore be regarded as museums of ancient retroviruses. Phylogenetic studies have allowed these elements to be sorted into distinct groups that are usually termed “families.” Similar to artifacts in an archeological museum, time has left its traces on these retroviral elements and very often only “shards” of the once complete sequence remain. All known proviruses in the human genome have suffered from postinsertional mutations, deletions, and recombinations that render them non-infectious. Most often, only a single long terminal repeat (LTR) remained. In contrast to humans, several other species including mice and sheep carry infectious endogenous retroviruses (1–3).

The most recently integrated and best preserved human proviruses belong to a subgroup of the HERV-K elements dubbed HERV-K(HML-2). The HERV-K group can be divided into 10 families (4). The designation “K” comes from their use of a lysine tRNA to prime reverse transcription and human mouse mammary tumor virus like-2 (HML-2) indicates their relationship to the murine betaretrovirus mouse mammary tumor virus (MMTV). More than 90 proviruses of the HML-2 family are exceptionally well preserved and maintain open reading frames encoding functional viral proteins (4). These proteins are expressed and non-infectious particles are released in various cells, particularly in tumors and cancer cell lines derived from teratocarcinomas and melanomas (5–7). Moreover, two research groups have demonstrated that consensus sequences of HERV-K(HML-2) elements are able to produce infectious particles (8, 9). The infection and replication of these particles is inhibited by a variety of restriction factors including members of the APOBEC family, tetherin and most likely other, yet to be identified antiviral factors that have evolved over millions of years of coevolution between this virus and its primate hosts.

HERV-K proviruses can be classified into two types. Only type II proviruses express the accessory Rec protein that functionally resembles the HIV Rev and HTLV Rex proteins (10, 11). Type I proviruses have a 292-bp deletion that prevents expression of the *rec* gene but can instead express an alternative protein called Np9. Both Rec and Np9 are associated with malignancies. In addition to the evidence for the protein-induced or promoted tumorigenesis of somatic cells, HERV-K elements are also implicated in cancer development at the DNA or RNA level. In this context, HERV-K proviruses have been shown to be involved in recombination events that in one setting left an LTR-promoter closely upstream of exons 5–12 of the *ETV1* gene that encodes a transcription factor of the Ets family. Androgen-dependent expression of the protein fragment driven by the viral LTR was correlated with an increase in the invasiveness of prostate cancer (12). Promoters of other HERV elements and retrotransposons have also been shown to stimulate expression of nearby genes that promote malignancy (13). The number of known genes that are under definite transcriptional control by HERVs and our understanding of the mechanisms involved have dramatically increased in recent years (14).

In this regard it should be stressed that transcription of HERV-K and other HERV elements is usually suppressed by epigenetic factors such as DNA methylation and heterochromatin-silencing by histone modifications (15). Nevertheless, it is estimated that even in normal healthy cells, retroviral LTRs initiate 10 times as many transcripts as regular promoters (16–19). The transcripts induced include many RNAs that are complementary to regular gene transcripts and can downregulate the expression of cellular genes (17). During tumorigenesis (and also, presumably, in other poorly understood situations) this epigenetic control can be partially or selectively weakened and particular retroviral promoters become able to vigorously activate the transcription of cellular genes.

## Endogenization of Retroviruses with a Focus on HERV-K(HML-2)

The prerequisite for acquisition of a new endogenous retrovirus is infection of a germ line cell that results in integration of the proviral DNA into the cell’s chromosomal DNA. If this integration and the possible replication of this founder virus do not prevent fertilization, a fetus can form that carries the retroviral element in every one of its somatic and germ line cells. From a genetic perspective, the provirus becomes a regular host gene that is passed down the generations, being inherited in a Mendelian fashion (20–22). Such endogenization and transfer to offspring has been demonstrated in the laboratory by infection of fertilized eggs with recombinant avian leucosis virus (23). The viremia in the carriers can be attributed to replication of either the exogenous or the endogenous form, which results in repeated infection of somatic cells. It can be assumed that frequent recombination events occur at that stage by copackaging of vRNAs, one from an inherited endogenous element and one from an infecting exogenous virus with template switching by the reverse transcriptase (RT) occurring during subsequent infection of a new host cell. To some degree, germ cells might also be repeatedly infected, leading to further colonization of the germ line and amplification of the number of viral elements belonging to the same family. Other modes of amplification are also conceivable and have been documented, including retrotransposition or duplication of chromosomal regions that contain HERV elements (24). However, in the case of HERV-K(HML-2) it appears that the predominant mode of amplification within the human genome has been reinfection by circulating infectious viruses over a period of at least 30–40 million years (25). Colonization of the human genome by this betaretrovirus did not occur at the same frequency all the time: there were bursts of integration, during which the replicative activity of the virus is assumed to have been high, interspaced with longer periods of low integration (25). In recent years the number of known human-specific HERV-K(HML-2) loci has risen (4, 10, 26) and the lack of these loci in chimpanzees, our closest primate relatives, proves that elements of this family continued to reinfect our ancestors after their split from the chimpanzee lineage about five to six million years ago. Although there is no evidence for it being ongoing in humans, retroviral integration is not something that only occurred in the distant past. In Australia we are currently witnessing a fulminant endogenization process in koala bears involving a retrovirus presumed to have been acquired from a species of Asian mouse only a hundred or so years ago (27–29).

**Figure 1:**
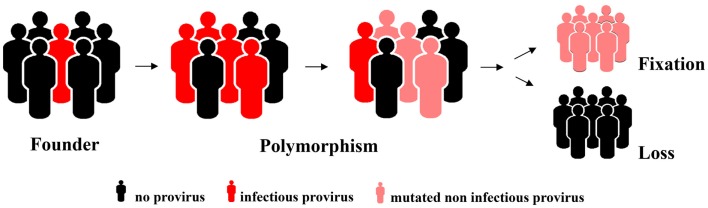
**Hypothetical model for an increase in allelic frequency and eventual loss or fixation of an endogenized element integrated at a particular chromosomal locus**.

The reproductive success of individuals carrying a germ line provirus at a particular chromosomal locus determines the frequency of this element in the population, i.e., it is subject to host selection (Figure 1). Selective forces and fluctuations in the population size (first and foremost bottlenecks that simply by chance increase the relative ratio of the carriers in the population) might eventually result in fixation of the element. This means it is present in all members of the population. It can be inferred that the likelihood of an element reaching fixation correlates negatively with its virulence if it compromises the reproductive success of the carrier. It is therefore likely that neutral elements or elements that even endow the carrier with a selective advantage are more likely to reach fixation whereas those with a negative impact will not and will eventually vanish. One piece of solid evidence in favor of this hypothesis is the orientation of the endogenous elements found in introns. Although integration has no preference with regard to direction, the majority of HERVs have an orientation opposite to that of the gene they have integrated next to, usually rendering the splice sites and other regulatory elements inactive. Elements that occurred in the same orientation as the gene vanished with higher frequency because they can modify expression of the encoded cellular protein to the detriment of the host. In contrast, there are only very few elements that are obviously under strong positive selective pressure, none of which belong to the HERV-K(HML-2) family (30).

## HERV-K Families

To date, there is little known about members of the HERV-K superfamily other than HERV-K(HML-2). All HML families belong to the class II/betaretrovirus group, which also contain the endogenous and exogenous MMTV, Mason–Pfizer monkey virus (MPMV), and Jaagsiekte sheep retrovirus (JSRV) (31). Elements such as HML-4 and HML-6, which are related to but clearly distinct from the HERV-K(HML-2) prototype virus HERV-K10 (32), were identified more than 20 years ago by extensive lab work using low-stringency Southern blot analyses with MMTV-derived *gag/pol* probes (33). These data were later complemented by PCR and the sequences were grouped into the HERV-K families HML-1 to HML-6, based on the sequences of a conserved RT region (34). These results were subsequently compiled to yield our current understanding that there are up to 10 HERV-K(HML) families in addition to a related family HERV-K(14C) present in the human genome (35). As the complete human genome has now been sequenced, identification of HERV elements can now be performed with relative ease by database analyses. Searching for sequences derived from the cloned HERV-K (HML-1 to HML-10) elements reveals a probable total of about 550 HERV-K proviruses and 6400 solitary LTRs in the human genome (36), ranging from 150 HML-3 proviral elements to 5 HML-4 proviruses, for example (24, 37). Not all HML families are described in detail using *in silico* generated consensus sequences or by analysis of individual elements, a summary of the features of HERV-K families is given in Table 1, although these numbers may need to be revised as was recently done for HML-2 solo LTRs, being adjusted from ∼2500 to 944 (4, 38).

**Table 1:** **Class II/HERV-K (human MMTV-like) families**.

Name	Alternative names	Estimated time of integration (mya)	Estimated copy number in genome		Reference
			Proviruses	Solo LTRs*	
HML-1	NMWV6, HERV-K14I	∼39	68	350	Franklin et al. (33), Medstrand and Blomberg (34), Flockerzi et al. (41)
HML-2	NMWV1, HDTV	∼35–2	91	944	Subramanian et al. (4), Bannert and Kurth (24), Ono (42)
HML-3	NMWV5, HERV-K9I	∼36	140	700	Franklin et al. (33), Medstrand and Blomberg (34), Mayer and Meese (43)
HML-4	HERVK13I, HERV-K-T47D	∼55–35	5	800	Medstrand and Blomberg (34), Seifarth et al. (37)
HML-5	NMWV2, HERV-K22I	∼55	139	600	Franklin et al. (33), Lavie et al. (40)
HML-6	NMWV4, HERV-K3I	∼30	30–40	50	Franklin et al. (33), Medstrand et al. (44), Yin et al. (45)
HML-7	NMWV7, HERV-K11D1	n.d.	20	140	Franklin et al. (33), Andersson et al. (35)
HML-8	NMWV3, HERV-K11I	n.d.	60	600	Franklin et al. (33), Andersson et al. (35)
HML-9	NMWV9	n.d.	10	40	Franklin et al. (33), Andersson et al. (35)
HML-10	HERV-K(C4)	∼30	10–50	100	Jern et al. (31), Tassabehji et al. (46)

* Most copy numbers of solo LTRs given in Ref (24); mya, million years ago; n.d., not determined.

The oldest group within the HERV-K superfamily appears to be the HERV-K(HML-5) family. Based on the traditional criterion for naming the HERV elements, HML-5 proviruses could constitute the HERV-M family, as the primer binding site of the consensus sequence is identical to the 3′ end of methionine tRNA. These elements are not found in prosimians, and are the only HERV-K proviruses in both Old World and New World monkeys, suggesting that the integration event must have taken place 35–55 million years ago. An even more precise estimate for the time of integration can be made by examining the LTRs. At that point in time, both LTRs in the viral genome would have been identical, but in the host genome they would accumulate random mutations at a rate of 2.3–5 × 10^−9^ substitutions per site per year (39), i.e., one difference between the two LTRs would be generated every 200,000–450,000 years. Although this kind of estimation has several limitations, including the fact that there were certainly periods of varying mutational rates, the integration of HML-5, based on LTR–LTR divergence, is estimated to have occurred ∼55 million years ago. It is interesting to speculate whether the age of HML-5 makes it the ancestor of other HERV-K families. However, sequence comparisons show that HML-5 does not lie at the root of the HERV-K phylogenetic tree, suggesting that this is probably not the case (40).

Remarkably, within the HML-1 family seven proviruses were found in which *gag*, *pro*, and *pol* sequences have been replaced by similar regions from HERV-W, resulting in hybrid structures (41). Such recombination events can occur by template switching by RT or at the DNA level. However, the differing flanking cellular sequences indicate that at some point the initial hybrid provirus was able to form progeny proviruses in the genome through a transcript.

## HERV Genes, Transcripts, Proteins, and Structure

**Figure 2:**
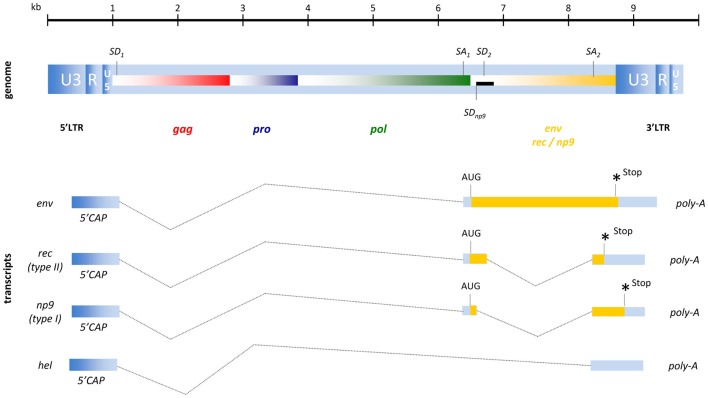
**Proviral organization of HERV-K(HML-2) and RNA transcripts**. The black bar represents a 292-bp deletion that results in the Np9 gene product for type I proviruses instead of the Rec (an HIV-1 Rev homolog) characteristic of the type II proviruses. *Hel* is a poorly described RNA without protein coding function.

To varying degrees, endogenous retroviruses are phylogenetically similar to exogenous retroviruses and it is therefore common to categorize them into three different classes. While class I HERV elements (e.g., HERV-W, HERV-FRD) have similarities to exogenous gammaretroviruses (e.g., MLV, GaLV) and class III HERV (e.g., HERV-L) are distantly related to lenti- or spuma-like retroviruses (e.g., HFV), the structure of a HERV-K(HML-1 to HML-10) element resembles a typical betaretrovirus (31). As HERV-K(HML-2) elements, in addition to the four major ORFs for *gag*, *pro*, *pol*, and *env*, also encode an accessory gene similar to those of delta-, lenti-, and spumaviruses they were defined as complex endogenous retroviruses. HML-2 type II proviruses encode an HIV-1 Rev- or HTLV-1 Rex-like protein termed Rec and only 26% of HML-2 elements are classified as type I expressing the protein Np9 (4, 47, 48). The genomic organization of these proviruses and their resulting RNA transcripts are shown in Figure 2.

The full-length HML-2 mRNA encodes the structural proteins of Gag, termed matrix (MA, p15), capsid (CA, p27), nucleocapsid (NC, p10) plus three smaller peptides as well as the putative phosphoprotein (pp15) (49). Due to two slippery sites and the resulting −1 bp ribosomal frameshifts, the Gag-Pro and Gag-Pro-Pol precursor proteins are produced and processed into the protease (Pro) enzyme and into polymerase (Pol) that exhibits RT and integrase (IN) activity. The single spliced mRNA for Env encodes the typical retroviral Env protein consisting of a signal peptide, a surface unit (SU; ∼44 kDa), and a transmembrane unit (TM; ∼26k Da). A functional Env protein has only been described for the HERV-K108 provirus (50), although the infectivity of another HERV-K(HML-2) element (K113) could be restored by back mutation of putative post-insertional amino acid changes (51). In addition to another two mRNAs coding for the accessory proteins Rec or Np9 that will be addressed in detail later, a 1.5-kb transcript termed *hel* has been found in human teratocarcinoma cell lines (48) although, as this mRNA does not encode a protein, its function requires clarification.

Some exogenous and endogenous retroviruses code for a dUTPase in addition to the enzymes essential for the retroviral life cycle such as RT, RNase H, Pro, and IN. dUTPase catalyzes the degradation of dUTP in order to prevent the toxic misincorporation of uracil into the newly forming proviral DNA. Both betaretroviruses and non-primate lentiviruses [i.e., Maedi-Visna virus (MVV) and equine infectious anemia virus (EIAV)] feature a dUTPase, albeit located in different regions of the genome (31, 52). The dUTPase in betaretroviruses is found within the N-terminal region of *pro* whereas in non-primate lentiviruses it is in *pol*.

## Postintegrational Mutations and Replication Potential of HERV-K

It can be assumed that unless antiretroviral cellular factors of the APOBEC-superfamily were deaminating their genomes during reverse transcription, the majority of HERV-K(HML-2) elements were replication competent for some period of time after integration into the germ line cell genome. HERV-K(HML-2) is particularly susceptible to APOBEC 3F and APOBEC 3G deamination as shown in infectivity assays using infectious viruses produced with HERV-K(HML-2) consensus sequences (9, 53). Moreover, 2 of 16 (53) most recently integrated elements known are substantially mutated by APOBEC deaminases, indicating that these antiviral factors were already active several millions of years ago. How long elements that escape restriction by deaminases are usually able to produce infectious viruses is an open question. It is known that some endogenous retroviruses of pigs, sheep, and other animals have resided in their host’s genomes for tens or even hundreds of thousands of years and yet retain open reading frames and remain both replication competent and infectious (21). Some replication competent porcine endogenous retroviruses in Old World pig species are presumably even older (3).

It is known that during their residency in the host cell genome, endogenous elements acquire postinsertional mutations and deletions that can inactivate the provirus. Recombination events frequently take place that delete most of the proviral sequence, leaving just an LTR (solo LTR). With HERV-K, solo LTRs outnumber elements still framed by both LTRs by a factor of at least 10. In contrast to these single LTR remnants, HERV-K(HML-2) proviruses also exist that possess open reading frames for all viral genes. Such elements include variants (haplotypes) of HERV-K113 and HERV-K108 that are thought to be two of the most recently acquired elements (4, 26). However, post-insertional mutations other than those that generate premature stop codons have since rendered these viruses replication incompetent (36, 54): infection could not be achieved using supernatants from germ line cells that release HERV-K(HML-2) particles. Indeed, none of the currently known HERV-K(HML-2) elements appear to have the capacity to produce infectious particles. It is not known when the most recent HERV-K(HML-2) endogenization occurred and when the last provirus lost its ability to replicate. In fact, although unlikely, the existence of an infectious HERV-K(HML-2) provirus with low prevalence in the current human population cannot be completely ruled out.

Even if no functional HERV-K(HML-2) element is currently extant in humans, an infectious virus might nevertheless be reborn as a chimera of partially inactivated parental elements. That infectious retroviruses can form by recombination between the RNA genomes of two inactivated parental elements is well documented, with the two most recent occasions involving murine gammaretroviruses, including the notorious XMRV (55, 56). Similar events could also result in the reconstitution of a replication competent HERV-K(HML-2) element. Indeed, Heidmann and Co-workers have demonstrated *in vitro* the reconstitution of an infectious HERV-K(HML-2) element through recombination of three HERV-K(HML-2) elements (8). Compared to exogenous retroviruses, the infectivity of such reconstituted particles is rather poor and productive replication in cell culture has yet to be demonstrated. It is therefore likely that despite the demonstration of *in vitro* infection and integration, restriction factors including APOBEC have evolved to prevent productive replication in cell culture and *in vivo*. However, HERV-K(HML-2) does not appear to be a target of human Trim5alpha, an antiretroviral factor attacking the capsids of infecting retroviruses (9).

## Role of HERV-K in Human Evolution and Physiology

In addition to the examples described in more detail below, transposable elements, including HERVs, generally drive alterations of the human genome by providing RT, an enzyme that can facilitate gene duplication for example, and by the addition or modulation of promoter activities. Even before the human genome was sequenced, it was suggested that constant updating of the transcriptional pattern through evolution of *cis*-acting elements might contribute more to species diversity than do changes in coding regions themselves (57). The simple insertion of an LTR can replace the original promoter, alter tissue specificity, or add enhancer activity (16). Of course, the LTRs acting as mobile genomic elements originate from full-length, inserted retroviruses.

Hypothetically, there are three possible outcomes of retroviral host genome invasion, i.e., genetic modification by the foreign DNA and/or the impact at the specific point of integration can be beneficial, harmful, or neutral for the individual. Retroviral integrations that severely harm the host genome, for example by interfering with the regulation of gene expression through promoter activity or direct destruction of coding regions, cannot play a significant role in human evolution because affected individuals are subject to negative selection and are removed from the gene pool. On the other hand, positive effects of altered gene expression through retroviral integration are well known. For example, the *amylase* gene is expressed in the pancreas of all mammalian species. However, in primates, rodents, and lagomorphs (rabbits, hares, and pikas), *amylase* is also expressed in the salivary gland as a result of gene duplication and insertion of a retroviral sequence (58), an event that probably helped these species to include starch in their diets.

Perhaps the most striking example of mammalian exploitation of retroviral sequences is the co-option of an endogenous retrovirus envelope gene to form the syncytiotrophoblast during pregnancy. The main function of the retroviral envelope protein is to mediate fusion of virus and target cell membranes during infection, making it an ideal “tool” to facilitate cell–cell fusion. The HERV-W (59) and HERV-FRD (60) glycoproteins, expressed exclusively in the placenta, are therefore termed *syncytin-1* and *-2*, respectively. Furthermore, the envelope proteins are involved in retrovirus-induced immunosuppression (61) and as the placenta, as far as the mother’s immune system is concerned, is an allogeneic organ, local immunological tolerance is necessary to prevent rejection of the fetus. This capacity for immunosuppression, shown for *syncytin-2*, but so far not for *syncytin-1* (59), may therefore be another reason that the endogenous retroviral *env* is expressed in the human placenta.

Further evidence for the deliberate co-option of retroviral genes during host evolution includes the presence of *syncytin-A* and *-B* genes in the murine genome, genes that have the same functions as their human counterparts but are clearly distinct at the sequence level (62). With mice it is possible to directly study the impact of these genes and “knocking out” *syncytin-A* results in disrupted placental architecture and the *in utero* death of embryos (63). The later discovery of *syncytin* genes in other mammals demonstrates that the exploitation of retroviral *env* genes has occurred independently and on multiple occasions throughout mammalian evolution. It is exciting to speculate that the first retroviral *env* integration in an oviparous species marked the beginning of the change from egg-laying *prototherians* to placental mammals (64).

A more direct advantage for the host of endogenization may be protection from infection by related exogenous pathogenic retroviruses. One well-studied example of this is sheep that carry an endogenous form of JSRV and are therefore protected against infection by exogenous JSRV (65) through receptor interference by the endogenous retroviral envelope. Furthermore, the Gag protein of defective transdominant endogenous JSRV interferes at a late stage of replication with the exogenous form of the protein (66), a phenomenon also described in mice in which expression of an endogenous retroviral Gag protein with homology to the HERV-L family blocks various strains of exogenous MLV (67). To date, no comparable function for HERV-derived proteins is known, but it is very plausible that the open reading frames retained for some HERVs serve a similar purpose.

## Expression and Reactivation of HERV-K Loci in Health and Disease

Although HERV-K(HML-2) gene expression is largely repressed in most cells by epigenetic silencing, low-level expression occurs in some tissues. Various groups have found that between 7 and 30% of all HERV sequences in the genome are transcriptionally active (18, 68, 69). In particular, the testes and placenta appear to be privileged tissues for HERV expression (69). There is an ongoing and active debate about HERV expression in healthy tissues, with some authors assuming a possible advantage for cells that express HERV-K proteins (70). Expression of the HERV-K envelope protein TM subunit in placental cytotrophoblast cells suggests its potential involvement in placentogenesis and pregnancy (71), as discussed above for *syncytin-1* and *-2*. Moreover, Wang-Johanning and Colleagues have detected low-level expression of HERV-K *env* mRNAs and proteins in healthy ovarian tissues (72).

Andersson and Colleagues made the very interesting discovery of HERV-K *rec* transcripts in the placenta and in various other normal human fetal tissues, suggesting that this small RNA transport molecule may play a role in the development and differentiation of human tissues (73).

Although HERV-K is expressed at only very low levels (if at all) in healthy tissues, activation of transcription, and protein production is associated with diseases such as cancer and autoimmune disorders. Indeed, the possible role played by upregulation of HERV-K expression in autoimmune diseases such as multiple sclerosis (MS) and rheumatoid arthritis (RA) is being investigated.

Rheumatoid arthritis is a chronic, systemic inflammatory disorder that especially affects synovial joints. Serological and molecular assays show a significant increase of HERV-K *gag* expression in RA patients compared to inflammatory and healthy control groups (74). Furthermore, exogenous viral protein expression and proinflammatory cytokines enhance HERV-K *gag* transcription. Finally, anti-HERV-K antibodies with possible cross-reactivity to host proteins could cause autoimmune responses through molecular mimicry (74).

Multiple sclerosis is more controversial, and although the etiology of this disease remains unknown it is likely that the trigger for MS is multifactorial. One interesting hypotheses involves a neurotropic infectious agent, possibly a virus, and HERV-K(HML-2) is one candidate in this context (75). Indeed, Tai and Colleagues found HERV-K18 expressing a superantigen in its *env* gene to be a risk factor for MS (76) and although this observation was made in a very small cohort, it is exciting because expression of HERV-K18 superantigen is also elevated in juvenile RA (77). There is also some evidence that, depending on the population, the LTR sequences of HERVs such as HRES-1 show polymorphisms within MS patient haplotypes while those from non-MS patients are identical without geographic restrictions (78). However, Moyes and Colleagues have published data showing that HERV-K113 and HERV-K115 are not significantly increased in patients with MS compared to the combined parent group (79, 80) and their results do not therefore support an association between these particular endogenous retroviruses and MS (79, 81). Finally, various groups have reported an association between HERV-K18 polymorphisms and type 1 diabetes (82, 83). In summary, a correlation between HERV-K expression and autoimmune disease is neither refuted nor proven. A definite conclusion will need to be based on a great deal more data.

In contrast to the situation with autoimmune diseases, the picture concerning HERV-K expression in malignant diseases is much clearer. As already mentioned, the expression of HERV-K(HML-2) in malignant tissues such as germ cell tumors (GCTs), melanomas, or ovarian cancers is considerably higher than in healthy tissues (5, 6, 70, 72, 84–86). A number of malignant diseases suggested to be associated with HERV-K expression are listed in Table 2. HERV-K expression was first observed in GCTs (87–89) and is often associated with the release of HERV-K(HML-2) particles. The frequent induction of an immune response to HERV-K(HML-2) Gag and Env proteins (90, 91) is well documented in patients with GCT disease. The presence of antibodies specific for HERV-K(HML-2) proteins correlates strongly with the clinical manifestation of GCTs and may therefore be used as a diagnostic and prognostic marker for these types of cancer (92). Interestingly, HERV-K expression in teratocarcinoma cell lines can be further enhanced by androgens (93), although, it is still unknown whether the androgen receptor (AR) binds directly to HERV-K LTRs or acts via other host proteins. Direct binding of other cellular proteins and transcription factors to the HERV-K(HML-2) LTR have already been demonstrated (94, 95).

**Table 2:** **List of malignant diseases that are associated with HERV-K(HML-2) activity**.

Tissue	Cancer	HERV-K(HML-2) activity	Reference
Skin	Melanoma	Retroviral particles	Buscher et al. (6), Muster et al. (86), Hirschl et al. (124)
		Enhanced transcription	
		RT activity	
		Expression of Env, Rec, Np9	
Testes	Germ cell tumors, gonadoblastoma, seminoma	Anti-Gag/Env-Ab	Boller et al. (87), Kleiman et al. (92), Boller et al. (125)
		Expression of Rec, Np9	
Ovary	Ovarian clear cell carcinoma; ovarian epithelial tumors	Expression of Gag and Env	Gotzinger et al. (15), Wang-Johanning et al. (72), Iramaneerat et al. (126)
Breast	Breast cancer	Free viral RNA	Wang-Johanning et al. (112), Contreras-Galindo et al. (127), Wang-Johanning et al. (128)
		RT activity	
		Virus particles	
		Specific CTLs	
Prostate	Prostate cancer	Enhanced Gag-production due to fusion to androgen-dependent ETV1 and ETS genes	Tomlins et al. (12), Lamprecht et al. (13), Ishida et al. (111)
Blood	Lymphoma	Free RNA	Contreras-Galindo et al. (127)
		RT activity	
		Virus-like particles	

Beside teratocarcinomas HERV-K proteins are expressed in 90% of epithelial ovarian tumors but at only very low levels, if at all, in healthy tissues or benign tumors (72). Activation of HERV-K expression in ovarian cancer might occur in response to stimulating transcriptional factors that are expressed in malignant ovarian cells (96). Such mechanisms, which lead to hypomethylation and subsequent enhancement of HERV-K transcription, have been already described for HERV-H and HERV-W elements in the ovaries (97–100) and for HERV-K in urothelial cancer and GCTs (101, 102).

Expression of HERV-K in melanomas has been the subject of intense study by many groups (6, 86). Both mRNA and Gag, Env, Rec, Np9, and RT proteins have been detected in specimens from primary and metastatic melanoma biopsies and melanoma cell lines but not in melanocytes or normal lymph nodes (6, 86). Muster and colleagues even observed retrovirus-like particles consisting of mature forms of Gag and Env and having RT activity (86). About 85% of malignant melanocytes express HERV-K MEL that is produced by a pseudo-gene incorporated into the HERV-K *env* gene and which has already been defined as a marker for melanoma risk (103–105). In accordance with the high levels of HERV-K expression, antibodies specific for Env and Gag were detectable in the sera of 16–22% of melanoma patients but not in those of healthy controls (6, 106). Hahn et al. showed in 2008 that the immune response of melanoma patients against HERV-K gene products correlates negatively with their survival (106). In line with existing knowledge about melanoma development, HERV-K expression can be triggered by UV radiation and may therefore be able to accelerate carcinogenesis (107, 108).

In addition to teratocarcinomas, ovary tumors, and melanomas, elevated levels of HERV-K(HML-2) *env* RNA were also detected in breast cancer tissues and *gag* RNA was found in the peripheral blood cells of leukemia patients as well as in breast and prostate cancers (109–112). The possible influence of steroid hormones on HERV-K activity in both breast cancer and prostate cancer is being discussed, with various groups reporting a stimulating effect of estroprogestin on HERV-K *env* transcription in breast cancer cells (110, 112, 113).

It was reported in early 2006 that, in contrast to patients undergoing successful HAART or to seronegative controls, HERV-K(HML-2) RNA can be found in the plasma of HIV-1 infected individuals whose antiretroviral therapy fails to control virus replication (114, 115). Subsequently, it was even possible to observe virus-like particles by electron microscopy and by immunostaining using HERV-K Env and Gag specific antibodies (116). A possible mechanism for preferential HERV-K expression in HIV-1 infected cells could be the interaction between HIV-1 Rev and the Rec response region in HERV-K(HML-2) RNA (11, 117) as well as the recently demonstrated susceptibility of the HERV-K (HML-2) transcriptional promoter to HIV-1 Tat (118).

The putative association between HERV-K(HML-2) expression and HIV-1 infection is strengthened by the detection of HERV-K specific cytotoxic T-cells in HIV-1 infected individuals (119). Interestingly, such HERV-specific T-cells are not found in HTLV-1 infected individuals (120), although this latter study cannot completely rule out an involvement of HERV activation in HTLV-1 pathogenesis. HERV-K specific CD8+ T-cells that can recognize and kill cells presenting the cognate peptides as a result of HIV-induced expression of HERV proteins (119) may play an important part in the natural immune response to HIV (121). In contrast to HIV specific CD8+ T-cells that are compromised in their ability to cope with the profound diversity of HIV-1 and subsequent appearance of escape mutants, the HERV proteins remain unchanged throughout the course of infection. Having an unchanging target allows HERV-specific T-cell clones from HIV-1 infected individuals to kill, *in vitro* at least, human CD4+ cells infected with viruses as diverse as HIV-1, HIV-2, and SIV (122). The stimulation of HERV-specific T-cells may therefore offer a new approach to inducing a broadly specific anti-HIV immune response. Of course, this approach is currently hampered by our limited understanding of HERV antigen expression in different individuals, tissues, and diseases (123).

## Implications for Cancer Development

Whether HERV-K expression is an initiator of cancerous processes or is simply a consequence of malignant degeneration remains a topic of discussion. Many groups have found evidence for the involvement of HERV-K in tumor processes and various hypotheses concerning its potential contribution to oncogenesis have been postulated. These possible mechanisms include the misregulation of oncogenes, protooncogenes, or growth factors by HERV-K LTRs or HERV loci. Several examples of transcriptionally active HERV loci that influence neighboring genes by providing alternative promotors are known (13, 129). Hypomethylation often affects HERV-derived solo LTRs that function as alternate promoters, for example the HERV-derived LTR is hypomethylated in B cell-derived Hodgkin’s lymphoma and is responsible for deregulated expression of the colony-stimulating factor 1 receptor (CSF1R), a protooncogene (13). Another possible mechanism for HERV loci to affect cellular gene transcripts is by the provision of alternative splice and polyadenylation sites [reviewed in Ref. (16)].

A second mechanism of tumor induction/promotion by HERV-K is the inactivation of tumor suppressor genes by *de novo* insertion or translocation of retroelements within the genome (70, 130). Recombination involving HERV-K sequences might also lead to chromosomal instability and large-scale chromosomal anomalies such as those typically observed in aberrant cells (131, 132).

A third mechanism is the expression of HERV proteins such as Rec and Np9 that function as oncogenes or of proteins that induce cell–cell fusion such as Env. It is possible that cell fusions initiated by Env contribute to tumor progression or might even play an important role in metastasis (133). Furthermore, Env is considered to have immunosuppressive properties and may therefore play a role in tumor escape processes (133–135). HERV-K may trigger the pathological processes that lead to melanoma development and contribute to the cellular modifications implicated in melanoma maintenance and progression (96, 136). UV radiation, hormone action, or mutating viruses lead to enhanced expression of HERV-K proteins such as Env or the HERV-K MEL-antigen that is particularly expressed in melanomas (96, 107, 137). The HERV-K Env protein is a molecular mimic of the oxygen responsive element binding protein (OREBP), a human nuclear factor that controls the expression of glutathione peroxidase, resulting in reduced production of the enzyme (137). This further increases the toxicity of free radicals that are no longer under proper control and enhances the risk of cancer.

## Potential Oncogenes: Rec and Np9

In recent years evidence has accumulated to indicate a role for the accessory HERV-K(HML-2) protein Rec and for Np9 in tumor development. Rec is a 14.5-kDa protein, translated from a doubly spliced transcript (Figure 2), that mediates the transport of unspliced or partially spliced HERV-K(HML-2) mRNAs from the nucleus into the cytoplasm (11). In 2005, Galli and Co-workers demonstrated that transgenic mice expressing Rec show disturbances in germ cell development and that some animals develop lesions similar to the predecessor lesions of classical seminomas in humans (138). This indicates that HERV-K(HML-2) Rec expression might play a direct role in cell transformation, creating a pre-cancerous state, although the molecular mechanisms and pathways involved remain elusive.

The cellular interaction partners of Rec are also poorly defined, although some have recently been identified. Rec interacts with the promyelocytic leukemia zinc-finger protein (PLZF) that is essential for the self-renewal of spermatogonial stem cells in mice and which may also be involved in certain human leukemias (139–142). Transient co-expression of Rec with PLZF interferes with its function as a negative regulator of the *c-myc* protooncogene and cells stably transfected with PLZF and Rec show increased cell proliferation (142). Recently, Kaufmann et al. reported that Rec forms trimeric complexes with the testicular zinc-finger protein (TZFP) and the AR (143). These data support the findings of other groups, including our own. Rec interacts directly with the human small glutamine-rich tetratricopeptide repeat-containing protein (hSGT), a co-chaperone and negative regulator of the AR (93, 144). Binding of Rec to the co-chaperone abrogates its ability to bind to the AR and to maintain it in an inactive state (93), which in turn leads to enhanced levels of AR activity. It is interesting that HERV-K transcription (and subsequently Rec expression) can be stimulated by hormones, including androgens, and various steroid hormone-binding sites are predicted to be located within the HERV-K LTR (89, 93, 95, 113).

In contrast to Rec, Np9 has no known physiological function in HERV-K replication. This 9 kDa protein is produced by viruses lacking a 292-bp sequence within the *pol-env* boundary. Np9, whose first 15 amino acids are identical to Env and Rec, is expressed exclusively in various tumor tissues such as breast cancer, GCTs, ovary cancer, and leukemia (47, 145). It is therefore important to identify host proteins potentially interacting with Np9.

Np9, like Rec, binds to the PLZF protein, albeit in a different region. Also like Rec, Np9 is able to abrogate the regulator of the *c-myc* protooncogene, leading to increased *c-myc* transcription that in turn leads to enhanced cell growth and reduced apoptosis (142).

Np9 has also been shown to interact with the ligand of Numb protein X (LNX), a RING-type E3 ubiquitin ligase that ubiquitylates Numb and marks it for degradation by 26S proteasomes (146). Numb is an antagonist of the transcription factor Notch and both proteins regulate the pro-proliferative Ras signaling cascade. Misregulation of this system has been linked to breast cancer, leukemias, and GCTs (147–151). Furthermore, Chen and Co-workers recently reported that Np9 is a potent viral oncogene in human leukemia (145). This small protein promotes growth of myeloid and lymphoblastic leukemic cells by activation of ERK, AKT, and Notch1 pathways and by upregulation of β-catenin. The latter is essential for survival of leukemia stem/progenitor cells.

## Concluding Remarks and Outlook

The examples of HERV activity described in this review make it clear that these elements are not simply pieces of “junk DNA” that litter the genome because a mechanism to completely remove an integrated retrovirus from the germ line does not exist. Despite having been discovered in the late 1960s, it appears that many of the intricate evolutionary and physiological roles in health and disease played by the retroviral part of our own genome still remain to be revealed. In addition, many questions concerning the endogenization process, the interplay of coexisting endogenous and exogenous forms, population dynamics, and the fixation of ERVs remain open. The regulation of human genes by HERV-K(HML-2) and other HERV families and the silencing and restriction mechanisms by which the host controls their activities are currently of particular interest.

From a virological point of view, the reconstitution of consensus sequences and expression of original HERV-K(HML-2) proteins have greatly facilitated studies into the functional role of viral proteins in the replication cycle and in cellular processes. This, as well as the increasing availability of whole genome sequences, significantly enriches the exciting and novel field of “paleoretrovirology.”

## Conflict of Interest Statement

The authors declare that the research was conducted in the absence of any commercial or financial relationships that could be construed as a potential conflict of interest.
